# Polymorphisms in *CLAUDIN1* and *SPINK5* Influence Skin Absorption of Pyrene, Pyrimethanil, and Oxybenzone in Human Volunteers

**DOI:** 10.1002/em.70050

**Published:** 2026-04-24

**Authors:** Emmy Keysendal, Gunnar Johanson, Lina Hagvall, Nanna Fyhrqvist, Christian Lindh, Karin Broberg, Emelie Rietz Liljedahl

**Affiliations:** ^1^ Division of Occupational and Environmental Medicine, Department of Laboratory Medicine Lund University Lund Sweden; ^2^ Institute of Environmental Medicine Karolinska Institutet Stockholm Sweden

**Keywords:** filaggrin, fungicide, polycyclic aromatic hydrocarbon, skin barrier, systemic exposure, ultra‐violet absorber

## Abstract

Absorption of chemicals through the skin affects occupational and environmental exposure to diverse compounds. We previously showed that loss‐of‐function (null) mutations and low–copy number variants (CNV) of *Filaggrin* (*FLG*), which encodes a key skin barrier protein, increased dermal chemical absorption; however, the *FLG* genotype did not explain all the observed variation. Here, we explore the effects of variation in genes encoding skin proteins that could affect chemical uptake. In a dermal exposure test, 23 null‐*FLG* and 31 wild‐type carriers were exposed to three common organic compounds: the polycyclic aromatic hydrocarbon pyrene, the fungicide pyrimethanil, and the ultraviolet‐light absorber oxybenzone. Liquid chromatography–mass spectrometry was used to measure the concentrations of these chemicals or their metabolites in the subjects' urine collected over a 40‐h period following exposure. We genotyped the participants for 14 polymorphisms in seven skin function‐related genes (*Filaggrin 2* [*FLG2*], including a new method for assessing *FLG2* CNV, *claudin 1* [*CLDN1*], *serine peptidase inhibitor kazal type 5* [*SPINK5*], *S100 calcium binding protein A7* [*S100A7*], *transmembrane protein 79* [*TMEM79*], *laminin subunit alpha 3* [*LAMA3*], and *involucrin* [*IVL*]) and performed a population toxicokinetic analysis. While controlling for *FLG* genotype, the *CLDN1* rs893051 minor allele was associated with increased absorption, faster absorption rate, and longer lag time, while the *SPINK5* rs2303067 minor allele was associated with shorter lag time. However, the differences in total systemic absorption were minor compared with *FLG* variants. Thus, *FLG* remains the predominant genetic determinant of chemical uptake through the skin.

## 1 Introduction

The skin is a complex structure that contains various proteins involved in a variety of vital interconnected processes that maintain skin integrity; however, our understanding of the genetic elements involved is limited. Filaggrin (FLG) forms a key component of the dermal barrier and is encoded in the human genome by *Filaggrin* (*FLG*). Translation of the *FLG* transcript produces pro‐filaggrin, which is then proteolytically processed to produce FLG.

Loss‐of‐function (null) mutations of *FLG* have been linked to skin barrier diseases, such as ichthyosis vulgaris, atopic dermatitis (Smith et al. 2006), and eczema (Sandilands et al. 2007). A human exposure study (Rietz Liljedahl et al. 2021) found that the *FLG* null genotype increased the dermal uptake of three common chemicals: the polycyclic aromatic hydrocarbon pyrene, the fungicide pyrimethanil, and the ultraviolet‐light absorber oxybenzone, selected due to their relatively low toxicity within their substance class. Additionally, humans show copy number variation (CNV, that is, variation in the number of repeats within the locus that encodes pro‐filaggrin) for *FLG*, and fewer copies of *FLG* were associated with faster dermal absorption rates, shorter lag times, and higher uptake of these compounds (Rietz Liljedahl et al. 2021). Carriers of specific genetic variants may, therefore, given similar external conditions, be subjected to higher systemic exposure to hazardous chemicals than non‐carriers, potentially increasing the risk of adverse skin and systemic effects (Anderson and Meade 2014; Liljedahl et al. 2024). However, the *FLG* genotype could only explain approximately 30% of the observed variation in dermal absorption of the three chemicals (Rietz Liljedahl et al. 2021), indicating that other genetic variants in the skin barrier may affect the dermal absorption.

Other vital genes expressed in the skin include *transmembrane protein 76* (*TMEM79*)*/MATT*, involved in skin lipid metabolism (Saunders et al. 2013); *Laminin subunit alpha3* (*LAMA3*), encoding Laminin‐5, which anchors the epidermis to the dermis (Nishiyama et al. 2000); and *Serine peptidase inhibitor kazal type 5* (*SPINK5*), which regulates epidermal differentiation and desquamation (Descargues et al. 2005). Variants in these genes have been associated with atopic dermatitis (Dežman et al. 2017; Saunders et al. 2013; Stemmler et al. 2014), highlighting their functional importance for the skin barrier and dermal absorption. Additionally, FLG shares expressional and structural similarities with other S100 proteins, including Filaggrin‐2 (FLG2) and Involucrin (IVL) (Henry et al. 2012; Toulza et al. 2007; Wu et al. 2009), both of which have genetic variants associated with skin disorders such as peeling skin syndrome (Alfares et al. 2017; Cubillos et al. 2015; Pellerin et al. 2013; Pendaries et al. 2015; Trzeciak et al. 2020). *Claudin‐1* (*CLDN1*) encodes an intercellular anchor protein crucial for regulating the transport of water and other molecules through the skin. Its loss or mutation leads to epidermal barrier defects, which are associated with atopic dermatitis (Asad et al. 2016), psoriasis (Watson et al. 2007), and the ichthyosiform skin phenotype of Neonatal Ichthyosis‐Sclerosing Cholangitis (NISCH) syndrome (Hadj‐Rabia et al. 2004). Thus, the altered function or expression of the above‐mentioned genes, possibly through polymorphisms, may affect the function of the epidermal barrier and increase susceptibility to chemical exposure via the skin.

The aim of this study was to attempt to explain some of the remaining variation in skin absorption that the 2021 study could not (Rietz Liljedahl et al. 2021). We did this by exploring the effect of polymorphisms in genes encoding seven proteins related to the skin barrier (*FLG2*, *CLDN1*, *SPINK5*, *S100A7*, *TMEM79*, *LAMA3*, and *IVL*) on the dermal absorption of a polycyclic aromatic hydrocarbon, a fungicide, and an ultraviolet‐light absorber. This will enhance our understanding of the role of proteins beyond FLG in skin barrier function, which may help identify individuals susceptible to chemicals via dermal exposure.

## 2 Materials and Methods

### 2.1 Selection of Polymorphisms

Fourteen polymorphisms of seven genes were selected based on previous studies that indicated their associations with defects in the skin barrier (Asad et al. 2019; Cubillos et al. 2015; De Benedetto, Rafaels, et al. 2011; De Benedetto, Slifka, et al. 2011; Dežman et al. 2017; Di et al. 2009; Kato et al. 2003; Lan et al. 2011; Margolis et al. 2014; Mathyer et al. 2021; Ross‐Hansen et al. 2013; Saunders et al. 2013; Stemmler et al. 2014; Walley et al. 2001; Zhao et al. 2012). The allele characteristics, predicted consequences of mutations, and minor allele frequencies (MAFs) were derived from Ensembl (Ensembl Genome Browser 111, n.d.) and National Center for Biotechnology Information (n.d.) databases. Using the Ensembl Linkage Disequilibrium function, SNPs with a reported strong linkage disequilibrium (*D*′ > 0.8) were excluded. The polymorphisms identified are presented in Table 1.

**TABLE 1:** Characteristics of the polymorphisms included in the study.

Polymorphism ID (NCBI)	Protein	Gene	MAF^a^	Study MAF *n* = 54	eQTL (GTEx)^b^
rs12568784	FILAGGRIN‐2	*FLG2*	T = 0.146	T = 0.20	Higher expression
XM_011509531.2 (CNV)^c^	FILAGGRIN‐2	*FLG2*	N/A	Frequency = 0.212	N/A
rs893051	CLAUDIN‐1	*CLDN1*	C = 0.399	C = 0.41	Lower expression
rs9290927	CLAUDIN‐1	*CLDN1*	T = 0.126	T = 0.14	Higher expression
rs3732923	CLAUDIN‐1	*CLDN1*	A = 0.389	A = 0.41	Lower expression
rs17501010	CLAUDIN‐1	*CLDN1*	T = 0.162	T = 0.10	N/P
rs16865373	CLAUDIN‐1	*CLDN1*	T = 0.04	T = 0.04	N/P
rs3774032	CLAUDIN‐1	*CLDN1*	T = 0.2	T = 0.12	Lower expression
rs2303067	LEKT1	*SPINK5*	G = 0.46	G = 0.53	Higher expression
rs2303070	LEKT1	*SPINK5*	T = 0.08	T = 0.06	Lower expression
rs3014837	S100 CALCIUM‐BINDING PROTEIN A7/PSORIASIN	*S100A7*	C = 0.05	C = 0.05	N/P
rs6684514	TRANSMEMBRANE PROTEIN 79	*TMEM79*	A = 0.29	A = 0.27	Lower expression
rs2337187	LAMININ SUBUNIT ALPHA‐3	*LAMA3*	A = 0.33	A = 0.32	Lower expression
rs4845327	INVOLUCRIN	*IVL*	T = 0.1	T = 0.09	Lower expression

Abbreviation: N/A, not available.

^a^ MAF = minor allele frequency, European population (CEU), 1000 Genomes Project Phase 3 populations. (Ensembl). eQTL = expression quantitative trait locus (mRNA), derived from GTExPortal.

^b^ Transcript variant ID. NCBI, CNV = copy number variation, N/P = not present. No significant expression quantitative trait loci (eQTLs) were identified.

### 2.2 Study Participants and Samples for Genotyping

An a priori power calculation was performed to detect differences related to *FLG* genotype. This provided the basis for the number of participants recruited in the exposure experiment (Rietz Liljedahl et al. 2021). The polymorphisms included in the present study were selected based on their known or suspected relevance to skin barrier function, but no further power calculations were conducted for these variants as samples had already been collected.

Samples for this study were obtained from a human exposure experiment conducted as described in a prior publication (Rietz Liljedahl et al. 2021). In brief, consenting volunteers (*n* = 432) received a saliva sample collection kit (Oragene DNA OG‐500 kit; DNA Genotek, Stittsville, Canada) and a questionnaire to collect data on their occupation, tobacco use, and whether they have a nickel allergy (Rietz Liljedahl et al. 2021). The saliva samples were returned to the laboratory of Occupational and Environmental Medicine, Lund University, Lund, Sweden. DNA was extracted from the saliva samples using the prepIT‐2P extraction kit (DNA Genotek), according to the manufacturer's instructions.

All subjects were genotyped for the four most common *FLG* null mutations in the European population: three single‐nucleotide polymorphisms (SNPs) (R501X, R244X, and S3247X), and one mutation (2282del4) (Rietz Liljedahl et al. 2021). Of the 432 volunteers, 28 carried a *FLG* null mutation and, of these, 23 consented to participate in the exposure experiment. One wild‐type carrier per *FLG* null carrier was included as control and matched on age and sex. Additionally, 8 wild‐type carriers were included as controls to increase statistical power, without matching. The study finally included 22 men and 32 women. Re‐genotyping was performed to validate the first genotyping. Long‐range PCR was performed to amplify *FLG* for CNV genotyping, which was unsuccessful for three participants who had the wild‐type *FLG* genotype (Rietz Liljedahl et al. 2021). The exposure experiment (Rietz Liljedahl et al. 2021) was conducted with pyrene (MilliporeSigma, Burlington, Massachusetts, USA), pyrimethanil (MilliporeSigma), and oxybenzone (Toronto Research Chemicals, Toronto, Canada) (Table S1). Blood and urine samples were collected 10–30 min prior to exposure. Three solutions were prepared: 0.118 μmol/mL of pyrimethanil in ethanol (Millipore Sigma), 0.103 μmol/mL oxybenzone in ethanol (MilliporeSigma), and 0.025 μmol/mL of pyrene in 1:1 ethanol:acetone (MilliporeSigma). Using a pipette, 200 μL of each solution was applied to defined areas on the skin: 23.6 nmol dose of pyrimethanil to a 5 × 5 cm area along the right bicep brachii, 20.6 nmol of oxybenzone to a 5 × 5 cm area along the left bicep brachii, and 4.9 nmol pyrene to a 5 × 10 cm area on the right volar aspect. Pyrimethanil was applied first, followed by pyrene, and then oxybenzone. After drying, the exposed areas were covered with aluminium foil for 4 h. Following exposure, the areas were washed three times with 70% ethanol. Participants completed a questionnaire about their medical history of asthma, eczema, or allergies, and their arms were photographed. Blood samples were collected immediately post‐exposure and centrifuged at 2200 × *g* for 10 min for serum and plasma, stored at −80°C. All voided urine was collected by the participants during the 4‐h exposure, as well as over a 48‐h period post‐exposure and sent in a cooler bag to the laboratory and stored at 5°C.

### 2.3 Liquid Chromatography–Tandem Mass Spectrometry (LC–MS/MS) Analysis

The urine samples were analyzed for OH‐pyrimethanil, oxybenzone, and 1‐OH‐pyrene using liquid chromatography–tandem mass spectrometry (LC–MS/MS; QTRAP 5500 and 6500+; AB Sciex, Framingham, MA, USA) at the Division of Occupational and Environmental Medicine at Lund University according to Rietz Liljedahl et al. (2021). Briefly, 500 μL urine was added with isotopically labeled internal standards for all compounds and de‐conjugated using β‐glucuronidase/aryl‐sulfatase enzyme prior to analysis. The limit of detection (LOD) was 0.1 ng/mL for OH‐pyrimethanil and 0.2 ng/mL for oxybenzone and 1‐OH‐pyrene. The precision of the methods was between 4% and 13%. The laboratory participates in the G‐EQUAS interlaboratory program for 1‐OH‐pyrene and oxybenzone. Creatinine was measured to account for urinary dilution, which was analyzed at the Department of Clinical Chemistry, Lund.

### 2.4 *FLG2* CNV Genotyping

To develop the *FLG2* assay, two *FLG2* transcripts with differing numbers of repeat domains were identified in the NCBI database. A long‐range PCR was performed based on the *FLG* CNV method (Rietz Liljedahl et al. 2021) to amplify the regions spanning the repeated domains. The long‐range amplified products were separated using gel electrophoresis. A detailed method for *FLG2* CNV genotyping can be found in the Supporting Information.

### 2.5 SNP Genotyping

The selected SNPs were genotyped using TaqMan predesigned SNP assays (Thermo Fisher Scientific, Waltham, Massachusetts, USA), including primers and probes. The 5‐μL total reaction volume in each well of the PCR plate contained a 3‐μL aliquot of a vortexed and centrifuged solution containing TaqMan genotyping Master Mix, sterile water, primers, and probes, as well as 2 μL of 2 ng/μL DNA (or water for the negative controls). All reagents were purchased from Thermo Fisher Scientific. The quantitative PCR (qPCR) plate was centrifuged for 20 s at 2000 rpm, after which the real‐time qPCR was run on an Applied Biosystems PCR machine (7900HT; Thermo Fisher Scientific) with the following conditions: 95°C for 10 min followed by 45 cycles of 92°C for 15 s and 60°C for 90 s. For rs3732923, the PCR conditions were optimized with an increased extension temperature, as follows: 95°C for 10 min followed by 45 cycles of 92°C for 15 s and 62°C for 90 s. An allelic discrimination analysis was performed using the Sequence Detection System program version 2.4.1 (Thermo Fisher Scientific).

### 2.6 Toxicokinetic Analysis

#### 2.6.1 Area Under the Urinary Excretion Rate Curve

The area under the urinary excretion rate curve (AUC) was used to measure the total systemic exposure to the three studied chemicals and was calculated from the excreted amount over time using the trapezoid method (Excel 2010, Microsoft) (Rietz Liljedahl et al. 2021). Although urine samples were intended to be collected over 48 h, not all participants provided this over the full time period; therefore, the AUC was calculated based on samples obtained over a 40‐h period (0–40 h after exposure). One subject was excluded due to a lack of sample data. For oxybenzone, an additional subject was excluded due to their pre‐exposure baseline concentrations exceeding the test‐exposure levels.

A *FLG* genetic score was generated by first combining the *FLG* loss‐of‐function mutations and CNV to place alleles into three categories: *FLG* null (carrier of *FLG* loss‐of‐function mutation), low CNV (wild‐type *FLG* with 20–22 CNV repeats), or high CNV (wild‐type *FLG* with 23–24 CNV repeats). The *FLG2* genetic score was then defined as: homozygous low CNV, heterozygous, and homozygous high CNV. Three subjects with missing data regarding their *FLG CNV* genotype were excluded from the *FLG‐*adjusted analyses.

#### 2.6.2 Lag Time and Absorption Rate

**FIGURE 1:**
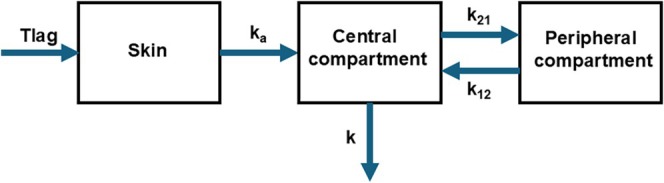
Overview of the compartmental model applied in the toxicokinetic analysis. The model includes a lag time for dermal absorption (*T*lag, h), a first‐order absorption rate constant through the skin (*k*_*a*_, h^−1^), the volume of the central compartment (L), transfer rate constants between central and peripheral compartments (k_12_ and k_21_, h^−1^), and a first‐order elimination rate constant from the central compartment (k, h^−1^).

Initial statistical analysis revealed indications of differences in AUC across CLDN1 rs893051 and SPINK5 rs2303067 genotypes and were accordingly selected for further toxicokinetic analysis regarding lag time and absorption rate. A first‐order, three‐compartment model (Figure 1) (Rietz Liljedahl et al. 2021) with skin absorption and excretion from the central compartment was used to evaluate the toxicokinetic behavior of the test chemicals. Dermal absorption was described by two parameters: lag time (*T*lag) and absorption rate constant (*k*_*a*_) (Figure 1).

In Monolix (version 2021R1; Lixoft, Antony, France), a statistical model built on the toxicokinetic model was used to evaluate dermal absorption by estimating lag time and absorption rate constant. The software combines an observational model that analyses the relationship between the measured variables with an individual model that accounts for variability across individuals. The observational model was built with distribution set as normal, a residual error model proposed by the software after the initial analysis, and a log‐normally distributed random effect applied to all model parameters.

For the individual model, correlations between variables were accounted for if proposed by the software after initial analysis. Dermally applied dose (23,600 nmol pyrimethanil, 20,600 nmol oxybenzone, 4940 nmol pyrene), the mid‐time point (h, i.e., time point halfway between two adjacent observations), and excretion rate (nmol/h) were the parameters included in the statistical model to estimate lag time and absorption rate. The SNP genotype was included both as a categorical variable and as a modifiable parameter for lag time and absorption rate constant. For the adjusted models, the continuous variables of age and body mass index (BMI) and the categorical variables of sex and *FLG* genetic score were added as additional parameters of lag time and absorption rate constant.

### 2.7 Statistical Analyses

In SPSS (Statistics 25; IBM, Armonk, New York, USA), Shapiro–Wilk tests were performed on the AUC data to assess their normality. A cross‐table analysis combined with Pearson chi‐squared analysis was conducted to identify associations between the genotypes. ANOVA/ANCOVA analyses were performed to compare log‐transformed AUC (0–40 h) data between the genotypes, with age, sex, BMI, and *FLG* genetic score as covariates. Differences in the estimated lag time and absorption rate constant and their correlation with the variant genotypes were investigated using linear regression in Monolix.

## 3 Results

### 3.1 Study Population Characteristics

The mean age of the study population was 39.1 years, and the mean BMI was 24.3 (Table 2). There was a higher proportion of women (59% of participants) than men.

**TABLE 2:** Study population characteristics (*n* = 54).

Characteristic	Value
Age (mean ± SD)	39.1 ± 15
BMI (mean ± SD)	24.3 ± 5
Sex (*n* (%))	
Female	32 (59)
Male	22 (41)
*FLG* polymorphism (*n* (%))	
*FLG* null	23 (45)
Low CNV: *FLG* WT 20–22	20 (39)
High CNV: *FLG* WT 23–24	8 (16)

*Note:* Data is presented as mean ± SD (standard deviation) or number (%). Three participants lacked information on *FLG* CNV genotype.

Abbreviations: CNV, copy number variation; WT, wild type.

### 3.2 Polymorphism Characteristics

The MAFs for all SNPs were largely comparable with those previously reported for the European population (Table 1). A cross‐table analysis in combination with Pearson chi‐squared tests did not indicate correlations between the polymorphisms, with the exception of the *FLG* and *FLG2* polymorphisms. There was complete linkage between *FLG2* rs12568784 and *FLG2* CNV (χ2 (4, *N* = 52) = 104.00, *p* < 0.001, *r* = 1.00, *p* > 0.001). *CLDN1* rs893051 and *SPINK5* rs2303067 were identified as expression quantitative trait loci (eQTLs); the minor allele of *CLDN1* rs893051 correlated with lower gene expression in the skin, while the minor *SPINK5* rs2303067 allele was more highly expressed in the skin (Table 1), compared with the major allele carriers.

### 3.3 Area Under the Excretion Rate Curve

**FIGURE 2:**
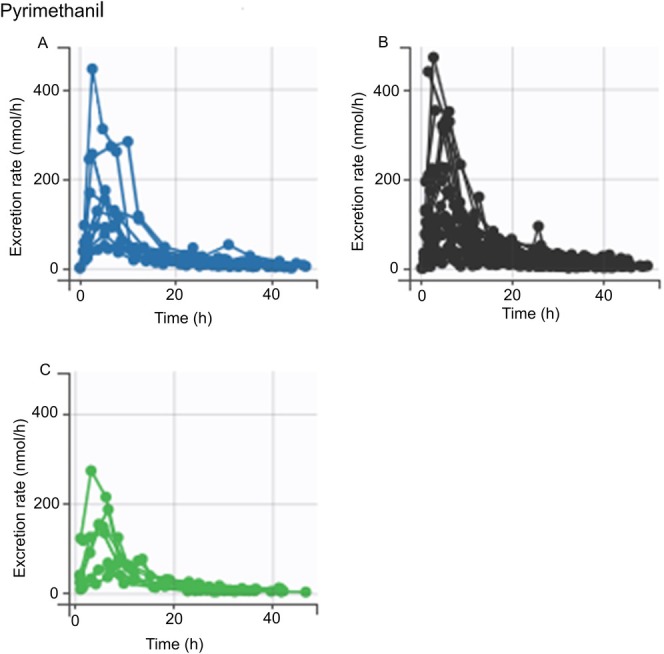
Pyrimethanil excretion stratified by *SPINK5* rs2303067 variant genotype. An excretion curve is plotted for each individual based on the excretion rate at the measured timepoints. (A‐C) Excretion curves of individuals with differing genotypes at *SPINK5* rs2303067. (A) Individuals homozygous for the minor genotype. (B) Individuals with the heterozygous genotype. (C) Individuals homozygous for the major genotype.

**FIGURE 3:**
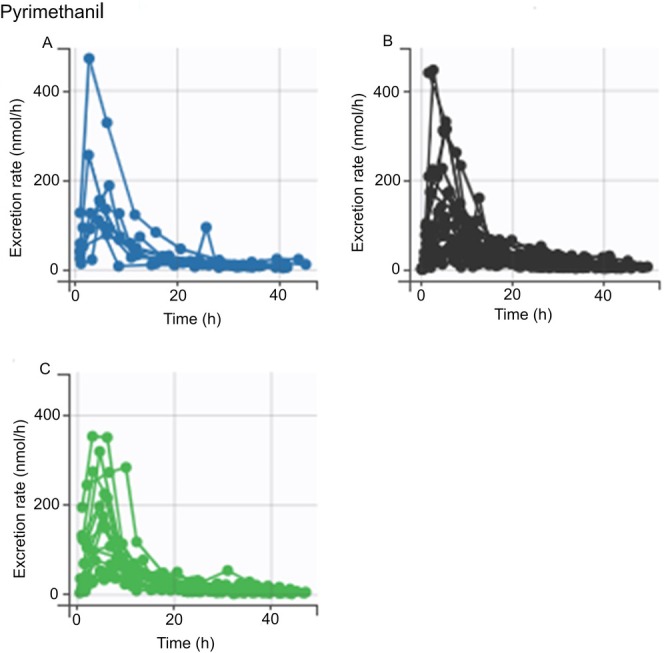
Pyrimethanil excretion stratified by *CLDN1* rs893051 variant genotype. An excretion curve is plotted for each individual based on the excretion rate at the measured timepoints. (A–C) Excretion curves of individuals with differing genotypes at *CLDN1* rs893051. (A) Individuals homozygous for the minor genotype. (B) Individuals with the heterozygous genotype. (C) Individuals homozygous for the major genotype.

**FIGURE 4:**
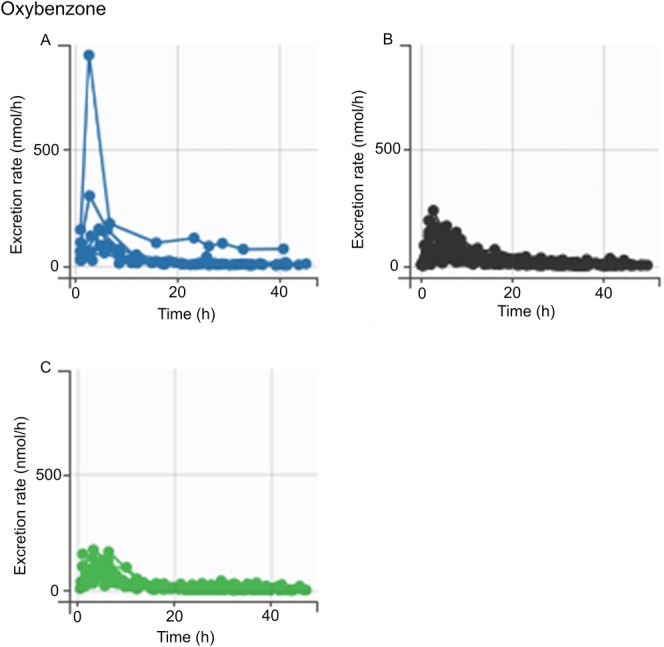
Oxybenzone excretion stratified by *SPINK5* rs2303067 variant genotype. An excretion curve is plotted for each individual based on the excretion rate at the measured timepoints. (A–C) Excretion curves of individuals with differing genotypes at *SPINK5* rs2303067. (A) Individuals homozygous for the minor genotype. (B) Individuals with the heterozygous genotype. (C) Individuals homozygous for the major genotype.

**FIGURE 5:**
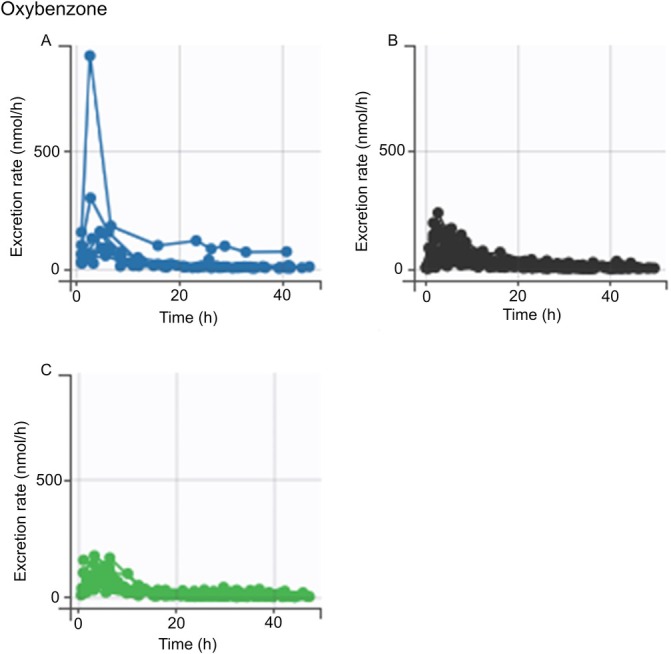
Oxybenzone excretion stratified by *CLDN1* rs893051 variant genotype. An excretion curve is plotted for each individual based on the excretion rate at the measured timepoints. (A–C) Excretion curves of individuals with differing genotypes at *CLDN1* rs893051. (A) Individuals homozygous for the minor genotype. (B) Individuals with the heterozygous genotype. (C) Individuals homozygous for the major genotype.

The ANOVA/ANCOVA analysis revealed no statistically significant differences in the AUC for any of the chemicals across the genotypes analyzed (data not shown). However, *SPINK5* rs2303067 heterozygotes and minor allele homozygotes appeared to have a higher AUC for pyrimethanil compared to the major allele homozygotes (Figure 2), but there were no statistically significant differences between genotypes, regardless of the adjustment of *FLG* genetic score (Tables 3 and 4). A similar trend was noted for *CLDN1* rs893051, although it was less pronounced (Figure 3; Tables 3 and 4). The excretion curves of oxybenzone did not show any genotype dependency for either of the variants (Figures 4 and 5), although one individual with homozygous minor genotypes for both variants displayed remarkably higher levels of oxybenzone excretion compared with the rest of the study population (Figures 4A and 5A). In the crude analysis, the pyrimethanil and oxybenzone AUC levels increased with increasing numbers of *CLDN1* rs893051 minor alleles, although this trend was not statistically significant (Table 3). However, the *CLDN1* rs893051 minor allele homozygotes showed statistically significantly higher oxybenzone AUC (1.7‐fold higher) than the major allele homozygotes when adjusted for *FLG* genetic score (*p* = 0.038; Table 4). The pyrene AUC did not display any clear trend across the genotype groups.

**TABLE 3:** Area under the urinary excretion curve (AUC_(0–40h)_), lag time, and absorption rate by genotypes of *CLDN1* rs893051 and *SPINK5* rs2303067.

Variant	Chemical	Genotype	AUC_(0–40h)_ (nmol, geometric mean; 95% CI)^a^	*p* (ANOVA)^b^	Lag time (h, mean ± SD)	Beta estimate, *p* ^c^	Absorption rate constant (h^−1^, mean ± SD)	Beta estimate, *p* ^c^
rs893051	Oxybenzone	Homozygous minor	1475.9; 917.42, 2727.3	0.186^d^	0.85 ± 0.1		0.41 ± 0.2	
		Heterozygous	1068.4; 841.7, 1362		0.19 ± 0.004	−41.4, < 2.2e‐16	0.17 ± 0.04	−7.68, 6e‐10
		Homozygous major	907.89; 709.59, 1158		0.3 ± 0.006	−26.6, < 2.2e‐16	0.24 ± 0.05	−3.91, 0.0003
	Pyrimethanil	Homozygous minor	1692.7; 1150.7, 2561.2	0.653^e^	0.82 ± 0.1		0.31 ± 0.07	
		Heterozygous	1461; 11,12.1, 1922.9		0.51 ± 0.07	−6.44, 4.5e‐8	0.2 ± 0.07	−3.4, 0.001
		Homozygous major	1288.5; 906.81, 1842.6		0.48 ± 0.03	−6.88, 9.3e‐9	0.23 ± 0.08	−1.85, 0.07
	Pyrene	Homozygous minor	27.7; 20.9, 36.4	0.4^f^	1 ± 0.2		0.078 ± 0.007	
		Heterozygous	28.6; 24, 34.1		1.2 ± 0.1	3.38, 0.001	0.072 ± 0.008	−1.74, 0.09
		Homozygous major	23.3; 17.6, 29.8		1 ± 0.1	−0.12, 0.9	0.076 ± 0.006	−0.44, 0.7
rs2303067	Oxybenzone	Homozygous minor	1189.9; 782.49, 1933.5	0.684^g^	0.57 ± 0.1		0.25 ± 0.1	
		Heterozygous	1002.3; 818.12, 1212.7		0.35 ± 0.02	−11.5, 1.5e‐15	0.26 ± 0.08	1.18, 0.2
		Homozygous major	1142.7; 821.47, 1519.3		0.62 ± 0.08	2.46, 0.02	0.37 ± 0.08	3.07, 0.004
	Pyrimethanil	Homozygous minor	1556.2; 1099.6, 2272.4	0.861^h^	0.32 ± 0.02		0.22 ± 0.07	
		Heterozygous	1419.9; 1100.5, 1848.8		0.5 ± 0.02	16.1, < 2.2e‐16	0.19 ± 0.07	−1.42, 0.2
		Homozygous major	1305.6; 894.02, 1919.2		0.78 ± 0.06	22.8, < 2.2e‐16	0.23 ± 0.07	0.089, 0.9
	Pyrene	Homozygous minor	26.5; 20.5, 34.1	0.933^i^	1 ± 0.2		0.078 ± 0.008	
		Heterozygous	26.2; 21.5, 31.3		1.1 ± 0.2	2.35, 0.02	0.072 ± 0.008	−2.11, 0.04
		Homozygous major	28.3; 24.5, 33.8		1.1 ± 0.1	1.62, 0.1	0.079 ± 0.005	0.55, 0.6

*Note:* The analysis is not adjusted for any covariates.

^a^ CI = confidence interval.

^b^ *p* values are derived from ANOVA (analysis of variance) for comparison between homozygous minor, heterozygous, and homozygous major.

^c^ Beta estimates and *p* values are derived from linear regression conducted in Monolix software. Beta estimate equals the regression coefficient, indicating the magnitude of change in AUC_(0–40h)_ per genotype relative to the homozygous minor genotype.

^d^ Adjusted *R* ^2^ = 0.028.

^e^ Adjusted *R* ^2^ = −0.022.

^f^ Adjusted *R* ^2^ = −0.003.

^g^ Adjusted *R* ^2^ = −0.025.

^h^ Adjusted *R* ^2^ = −0.034.

^i^ Adjusted *R* ^2^ = −0.037.

**TABLE 4:** Area under the excretion curve (AUC_(0–40h)_), lag time, and absorption rate by genotypes of *CLDN1* rs893051 and *SPINK5* rs2303067, adjusted for the *FLG* genetic score.

Variant	Chemical	Genotype	AUC_(0–40)_ (nmol, geometric mean; 95% CI)^a^	*p* (ANCOVA)^b^	Lag time (h, mean ± SD)	Beta estimate, *p* ^c^	Absorption rate constant (h^−1^, mean ± SD)	Beta estimate, *p* ^c^
rs893051	Oxybenzone	Homozygous minor	1475.9; 910.65, 2624.4	0.101^d^	0.74 ± 0.2		0.41 ± 0.1	
		Heterozygous	1039.2; 819.58, 1320		0.36 ± 0.1	−26.2, < 2.2e‐16	0.19 ± 0.06	−26.2, < 2.2e‐16
		Homozygous major	861.03; 656.71, 1118.5		0.26 ± 0.1	−41.8, < 2.2e‐16	0.25 ± 0.06	−41.8, < 2.2e‐16
	Pyrimethanil	homozygous minor	1692.7; 1151.6, 2651.4	0.401^e^	0.69 ± 0.06		0.22 ± 0.04	
		Heterozygous	1422.4; 1068.3, 1848.3		0.48 ± 0.1	−5.12, 6.3e‐6	0.15 ± 0.04	−2.52, 0.02
		Homozygous major	1224.1; 832.9, 1800.3		0.33 ± 0.05	−6.93, 1.3e‐8	0.17 ± 0.04	−1.77, 0.08
	Pyrene	Homozygous minor	27.7; 20.7, 36.7	0.391^f^	0.98 ± 0.2		0.079 ± 0.004	
		Heterozygous	27.6; 23, 32.7		1.1 ± 0.2	−0.36, 0.7	0.072 ± 0.006	−2.66, 0.01
		Homozygous major	23.7; 17.4, 31.7		0.97 ± 0.2	−0.25, 0.8	0.076 ± 0.004	−2.47, 0.02
rs2303067	Oxybenzone	Homozygous minor	1207.2; 763.1, 2022.2	0.489^g^	0.33 ± 0.2		0.26 ± 0.1	
		Heterozygous	958.23; 768.66, 1194.2		0.4 ± 0.2	6.67, 3.5e‐8	0.26 ± 0.09	1.97, 0.06
		Homozygous major	1142.7; 854.41, 1522.3		0.33 ± 0.2	9.86, 1e‐12	0.35 ± 0.1	4.57, 3.9e‐5
	Pyrimethanil	Homozygous minor	1569.5; 1067.4, 2222.4	0.876^h^	0.22 ± 0.03		0.2 ± 0.06	
		Heterozygous	1359.2; 1028.4, 1782.7		0.46 ± 0.07	23.5, < 2.2e‐16	0.19 ± 0.06	−0.26, 0.8
		Homozygous major	1305.6; 888.26, 1886.1		0.76 ± 0.1	31.3, < 2.2e‐16	0.23 ± 0.07	1.65, 0.1
	Pyrene	Homozygous minor	27.3; 20.7, 36	0.792^i^	0.98 ± 0.2		0.079 ± 0.004	
		Heterozygous	25.5; 20.9, 30.9		1.1 ± 0.3	1.59, 0.1	0.074 ± 0.004	−1.51, 0.1
		Homozygous major	28.3; 24.2, 33.9		1.2 ± 0.2	1.61, 0.1	0.075 ± 0.005	1.35, 0.2

^a^ CI = confidence interval.

^b^ *p* values are derived from ANOVA (analysis of variance) for comparison between homozygous minor, heterozygous, and homozygous major.

^c^ Beta estimates and *p* values are retrieved from the linear regression performed in Monolix, where they are deviations from the “homozygous minor”‐genotype estimates.

^d^ Adjusted *R* ^2^ = 0.167, statistically significant pairwise comparation between homozygous major and homozygous minor; *p* = 0.038.

^e^ Adjusted *R* ^2^ = 0.165.

^f^ Adjusted *R* ^2^ = 0.105.

^g^ Adjusted *R* ^2^ = −0.005.

^h^ Adjusted *R* ^2^ = 0.119.

^i^ Adjusted *R* ^2^ = 0.086.

### 3.4 Toxicokinetic Analysis

While there were no statistically significant differences in AUC, the *CLDN1* rs893051 and *SPINK5* rs2303067 polymorphisms exhibited trends suggesting potential genotype‐dependent variation in dermal uptake and were therefore subjected to a toxicokinetic analysis to further explore the lag time and absorption rate. Across the two variants, pyrene displayed the longest lag time and a slower absorption rate compared with oxybenzone or pyrimethanil. Within each polymorphism, the lag time and absorption rate were similar between pyrimethanil and oxybenzone (Tables 3 and 4).

For both variants, there were statistically significant differences (*p* < 0.05) in the pyrimethanil and oxybenzone lag time (between the minor allele homozygotes and the other genotypes), as shown in Tables 3 and 4. For pyrimethanil, SPINK5 rs2303067 minor allele homozygotes exhibited a shorter mean lag time than the other genotypes, while the minor allele homozygotes of CLDN1 rs893051 had a longer mean lag time. For CLDN1 rs893051, the minor allele homozygotes had a faster absorption rate for oxybenzone than the heterozygous and homozygous major allele genotypes, 120% higher and 65% higher, respectively. By contrast, the absorption rate of both chemicals was not dependent on the SPINK5 rs2303067 genotype. There were no significant associations between the lag time or absorption rate for pyrene for either of the variants. The effects were consistent between the crude and the FLG‐adjusted model, while further adjustments resulted in inflated and unstable estimates, likely due to insufficient statistical power (Tables S2 and S3).

## 4 Discussion

Of the 14 variants in the seven genes included in this study, *CLDN1* rs893051 and *SPINK5* rs2303067 showed some associations with the dermal uptake of oxybenzone and pyrimethanil. These two variants may affect skin absorption by influencing either lag time, as *SPINK* rs2303067 was associated with shorter lag time, or absorption rate, as *CLDN1* rs893051 was associated with a slower absorption rate. By contrast, pyrene demonstrated no genotype‐specific pattern, with consistently lower estimated internal dose (AUC), notably lower than what would be expected given pyrene's administered dose being approximately one‐fourth that of the other compounds. Although the three chemicals have similar molecular weights, pyrene has a significantly higher lipophilicity compared to oxybenzone and pyrimethanil (Table S1), and thus a larger deviation from the ideal lipophilicity of approximately logP = 2 for skin uptake (Cross et al. 2003; Karlberg et al. 2008). The high lipophilicity and rigidity of the polyaromatic ring system are likely to explain the low skin permeability of pyrene (Bo Nielsen et al. 2009; Potts and Guy 1992), indicated by the observed lower internal dose per applied dose, longer lag time, and slower absorption rate for this compound compared with the other tested compounds.

### 4.1 CLDN1

The *CLDN1* rs893051 minor allele homozygotes exhibited a higher internal dose of oxybenzone than the major allele homozygotes, suggesting that this variant plays a role in dermal absorption. Given the reduced expression of the minor allele (GTEx), one might expect impaired skin integrity in carriers, with increased dermal absorption as a consequence. Indeed, the observed faster absorption rate could be an explanatory factor for the increased systemic exposure associated with the minor allele. Nevertheless, the observed longer lag time associated with the minor allele indicates a slower stratum corneum absorption that would instead reduce dermal uptake. The biological function of *CLDN1* in paracellular diffusion is localized to the stratum granulosum layer (Furuse et al. 2002), suggesting that this variant may affect the absorption rate rather than the lag time, as lag time is primarily linked to the initial skin permeation of the stratum corneum. Our results demonstrate a statistically significant effect of the rs893051 allele on systemic exposure, as indicated by the AUC. This is consistent with previous studies identifying this allele as a risk factor for atopic dermatitis (Asad et al. 2016; De Benedetto, Rafaels, et al. 2011) and contact sensitisation (Ross‐Hansen et al. 2013), suggesting that carriers of this risk allele have altered skin integrity, potentially contributing to a higher dermal uptake of chemicals.

The *CLDN1* rs892051 major allele homozygotes exhibited a 42% reduction in the oxybenzone AUC relative to the minor allele homozygotes, after adjusting for the *FLG* genotype. We previously found that the wild‐type *FLG* 23–24 CNV genotype showed a 52% reduction in pyrimethanil AUC compared with the *FLG* null genotype (Rietz Liljedahl et al. 2021). This implies that dermal absorption of chemicals may be more influenced by the *FLG* genotype than the *CLDN1* rs892051 genotype.

### 4.2 SPINK5

The toxicokinetic analysis revealed a statistically significant association between a shorter pyrimethanil lag time and the *SPINK5* rs2303067 minor allele homozygotes. This aligns with the known function of SPINK5 as a serine protease inhibitor in the stratum corneum layer of the skin (Descargues et al. 2005), which could affect the initial absorption of chemicals. Furthermore, the eQTL analysis (GTEx) suggested that the minor allele carriers show higher *SPINK5* expression levels in their skin, which would be expected to inhibit the proteolytic activity and rescue skin integrity (Furio et al. 2015). This reasoning assumes an intact function of the encoded protein; however, previous studies of the *SPINK5* rs2303067 variant report induced epidermal proteolytic activity and profilaggrin processing associated with the minor allele (Fortugno et al. 2012). This may instead result in excessive degradation, compromised skin integrity, and increased chemical penetration, consistent with the observed shorter lag time associated with the minor allele. This further aligns with previous evidence linking the minor allele to skin defects (Dežman et al. 2017; Di et al. 2009; Kato et al. 2003; Walley et al. 2001; Weidinger et al. 2008).

In the present study, we did not identify any statistically significant differences in AUC between the genotypes of *SPINK5* rs2303067 for any of the chemicals, in contrast to the previously reported influence of the *FLG* genetic score (Rietz Liljedahl et al. 2021). This variant thus seems to have little influence on dermal absorption.

### 4.3 FLG2 CNV

We have introduced a new long‐range PCR method to determine *FLG2* CNV; the finding that *FLG2* rs12568784 is in complete linkage with the *FLG2* CNV genotype may be useful for further research, simplifying the genotyping of *FLG2* CNV.

### 4.4 Strengths and Limitations

The strengths of this study include the use of human subjects in an experimental model, which allows for a more specific evaluation of the selected chemicals in the complex nature of human toxicokinetics and toxicodynamics. Other strengths include the controlled dose and duration of the exposure experiment, as well as continuous urine sampling over a long post‐exposure period (48 h), enabling the specific measurement of the urinary excretion of the studied chemicals. Additionally, this study included the evaluation of multiple genes and polymorphisms related to skin barrier function.

A possible limitation of this research is that the study population was selected based on their *FLG* genotype. This could have caused selection bias if individuals with severe skin barrier defect symptoms declined to participate in a dermal experimental exposure study. Furthermore, this study was powered to detect differences related to *FLG* genotype, whereas the additional polymorphisms were included without specific power calculations. The sample size of 54 individuals may have limited the ability to detect true associations, particularly for variants with low minor allele frequencies. However, the majority of the polymorphisms included in this study had allele frequencies above 10%, which mitigates this concern to some extent. Nonetheless, replication in larger, independent populations is warranted. Further, the participants in the exposure experiment were selected from the Swedish population, and it should be noted that the variant allele frequency may vary across other populations with different ethnic origins.

The area under the curve (AUC) of urinary excretion rates for metabolites was used as an indicator of systemic exposure, based on its proportionality to absorbed dose. It should be acknowledged that urinary AUC is influenced by factors beyond systemic uptake, including metabolism, which vary between individuals. Nonetheless, for comparing relative exposure between groups, we consider urinary AUC as a relevant and practical proxy for systemic uptake. Metabolic rate and other toxicokinetic factors that may affect the AUC are unlikely to be linked to the investigated genotypes. Thus, variability in these factors is expected to mask rather than enhance differences between the genotypes.

The toxicokinetic model used in this project was created to estimate the lag time and absorption rate of the tested chemicals; however, the lack of early excretion data collection may cause the model to be less robust in accurately estimating these factors, which are the first aspects of the absorption process. The method could be improved by using other methods for more precise measurements of these parameters, such as the use of skin biopsies, rather than predictions from excretion data. On the other hand, taking skin biopsies is a more invasive procedure than the collection of urine samples and could impede enrollment in the study.

## 5 Conclusion

This study identified *SPINK5* and *CLDN1* gene variants that influence the absorption of chemicals through the skin. It also highlights the genetic complexity in the dermal uptake of chemicals and skin barrier function, while still emphasizing the *FLG* genotype as the main regulator of the total chemical exposure. Additionally, the *FLG2* SNP rs12568784 was found to be in complete linkage with the *FLG2* CNV genotype, allowing the optimization of *FLG2* CNV genotyping in future studies.

We conclude that the polymorphisms studied here, in addition to *FLG* polymorphisms previously investigated in this study population, may influence the dermal uptake of oxybenzone and pyrimethanil. Moreover, our findings indicate no association between the studied polymorphisms and systemic exposure. This adds to the notion that FLG functions as the key protein in dermal chemical uptake, given existing evidence of difference in systemic exposure by *FLG* genotype (Rietz Liljedahl et al. 2021).

## Author Contributions

K.B. conceptualized the study and applied for Research Ethics Board approval. K.B. and E.R.L. supervised data analysis and interpretation. C.L. collected the liquid chromatography – tandem mass spectrometry data from urine samples. E.K. performed genotyping analysis, statistical analysis, and drafted the manuscript. All authors participated in the revision of the manuscript and approved the final version. K.B. had complete access to the study data.

## Funding

The project was funded by the Swedish Research Council for Health, Working Life and Welfare (2016‐00700); the Swedish Environmental Protection Agency; the Swedish Research Council for Environment, Agricultural Sciences, and Spatial Planning; Karolinska Institutet, Region Skåne; and the Medical Faculty at Lund University.

## Conflicts of Interest

The authors declare no conflicts of interest.

## Supporting information


**Data S1:** em70050‐sup‐0001‐Supinfo.docx.

## Data Availability

The data that support the findings of this study are available from the corresponding author upon reasonable request.
