# Supplementary material for: Polymorphisms in CLAUDIN1 and SPINK5 Influence Skin Absorption of Pyrene, Pyrimethanil, and Oxybenzone in Human Volunteers
Source: Environ Mol Mutagen. 2026 Apr 24;67(3):e70050. doi: 10.1002/em.70050 (PMC13109691; doi:10.1002/em.70050)
Supplement: Supplementary file 1 — Data S1: em70050‐sup‐0001‐Supinfo.docx. [file EM-67-0-s001.docx]

**Supplemental Material**

**Polymorphisms in *CLAUDIN1* and *SPINK5* Influence Skin Absorption of Pyrene, Pyrimethanil, and Oxybenzone in Human Volunteers**

Emmy Keysendal^1^, Gunnar Johanson^2^, Lina Hagvall^1^, Nanna Fyhrqvist^2^, Christian Lindh^2^, Karin Broberg^1,2^, and Emelie Rietz Liljedahl^1*^

^1^ Division of Occupational and Environmental Medicine, Department of Laboratory Medicine, Lund University, Lund, Sweden

^2^ Unit of Integrative Toxicology, Institute of Environmental Medicine, Karolinska Institutet, Stockholm, Sweden

^*^ Address correspondence to Karin Broberg, Institute of Environmental Medicine (IMM), Box 210, 17177 Stockholm, Sweden. Email: karin.broberg@ki.se

1. *FLG2* CNV genotyping

1.1. Identification of CNV transcripts and primer design

The DNA sequence of *FLG2* was analyzed using a tandem repeat program (Benson, 1999) to define the repeating domains. Two different-sized *FLG2* transcript variants were identified (NCBI: NM_001014342.3, XM_011509531.2), with lengths of 9124 nt and 8690 nt, respectively. The transcript sequences were aligned using ClustalW Omega (Madeira et al., 2022) to detect differences in the presence and number of repeat domains. One variant was found to lack two A-domains in the third exon. The NCBI Primer-BLAST tool (Ye et al., 2012) was used to design primers flanking the 5′ portion of the third exon, encoding the A-domains. The forward primer was designed to complementarily bind to the second intron, while the reverse primer targeted the spacer sequence of exon 3 between the A- and B-domains. Amplification of the sequence encoding the B-domain was excluded to improve the amplification efficiency. The primer sequences were aligned to the whole genome using BLASTn (Altschul et al., 1990) to ensure high specificity for the *FLG2* gene.

1.2. Long-range PCR amplification of *FLG2*

Long-range PCR was performed as previously described (Rietz Liljedahl et al., 2021) although the annealing temperature was optimized for the new primers through test PCR runs with temperature gradients. The amplification produces different product sizes depending on the CNV. The total reaction volume was 15 μL, including 12 μL of a solution of sterile water (Thermo Fisher Scientific, Waltham, Massachusetts, USA), LaTaqM2+ buffer (10x; TaKaRa Bio, Kusatsu, Japan), dNTPs (2.5 mM; TaKaRa Bio), forward primer (10 μM 5′-TTCCACTCCTTCCTGTAGGC-3′), reverse primer (10 μM 5′-ACTTGTGGTTGGACCTGAGC-3′), and TaKaRa polymerase (5 U/μL;TaKaRa Bio); as well as 3 μL DNA or sterile water (negative control). The PCR was performed using a PCR machine (T100 Thermal Cycler; Bio-Rad Laboratories, Hercules, California, USA) with the following conditions: 94°C for 1 min, followed by 30 cycles of 98°C for 10 s, 62°C for 30 s, and 72°C for 8 min. The final extension step was performed at 72°C for 10 min.

1.3. Gel electrophoresis and CNV genotyping

The long-range PCR products were separated through gel electrophoresis on a 1% agarose gel prepared with 1x TAE buffer (50x TAE Buffer; Bio-Rad Laboratories, Solna, Sweden) and Gel Red Nucleic Acid Stain (10000X; BioTium, Fremont, California, USA). TAE (1x) buffer was used as the electrophoresis buffer. A 5-µL aliquot of PCR product was pipette-mixed with 2 µL 6x DNA loading dye (Thermo Fisher Scientific), after which 5 µL was loaded into the gel well. 1-kb Plus DNA ladder (Thermo Fisher Scientific) was loaded as a size reference. The expected product sizes were 3659 bp and 3203 bp (domain deletion), respectively. The running time for the gels used for genotyping ranged between 4 h 30 min and 5 h 20 min.

One gel was stained to ensure the DNA migration in the gel was optimized to minimize the disturbance of the nucleic acid stain and potential incorrect sizing of bands. In this method, the gel was cast (without the addition of the Gel Red Nucleic Acid Stain), loaded, and run according to the previously described protocol. Following the electrophoresis, the gel was incubated with gentle agitation for 30 min in 1x TAE with 3x diluted Gel Red Nucleic Acid Stain.

Gel bands were visualized under 302-nm UV light. Samples generating weak bands were re-run with 8 µL of the PCR product, while overloaded samples were re-run with 1 µL of the PCR product. For two samples, it was not possible to identify the *FLG2* CNV genotype. For four samples, two of each of the identified homozygous CNV genotypes, the gel bands were excised after electrophoresis and stored at –20°C for future sequencing.

2. Results

*FLG2* genotyping

The agarose gel bands revealed two different sizes of *FLG2* PCR products (Supplementary Figure 1), approximately 3800 bp and 3600 bp, respectively. The frequency of the domain deletion allele was 21.2% in the exposure study population (of the 52 participants, five individuals were homozygous and 12 were heterozygous). A cross-table analysis with Pearson’s χ^2^ test and a correlation coefficient analysis revealed a complete correlation between the *FLG2* SNP rs12568784 genotypes and *FLG2* CNV genotypes (χ^2^ (4, N=52) = 104.00, p<0.001, r=1.00, p>>0.001). Furthermore, a strong significant negative correlation was identified between the *FLG* score and the *FLG2* CNV genotype (χ^2^ (4, N=50) = 24.46, p>0.001), and rs12568784 genotypes (χ^2^ (4, N=51) = 25.03, p<0.001, r= –0.518, p<0.001). No significant correlations were found between the *FLG* score, *FLG2* CNV, or *FLG2* rs12568784 and any of the *CLDN1* SNPs. The genotypes of CLDN1 rs3732923 were positively correlated with rs893051 (Pearson’s χ^2^ (4, N=54) = 27.48, p <0.001, r=0.580, p<0.001) and rs17501010 (Pearson’s χ^2^ (4, N=52) = 12.08, p=0.017, r=0.217, p=0.123).

**Supplementary Table 1.** Chemical properties of chemicals used in exposure experiments.

| **Chemical name** | **CAS**  **Registry number** | **Molecular weight (g/mol)** | **Lipophilicity**  **(logP)** |
| --- | --- | --- | --- |
| Pyrimethanil | 53112-28-0 | 199.25 | 2.84 |
| Oxybenzone | 131-57-7 | 228.24 | 3.79 |
| Pyrene | 129-00-0 | 202.25 | 4.88 |

**Supplementary Table 2.** Area under the excretion curve (AUC_(0-40 h)_), lag time, and absorption rate by genotypes of rs893051 (*CLDN1*) and rs2303067 (*SPINK5*), adjusted for age, sex, and body mass index (BMI).

| **Variant** | **Chemical** | **Genotype** | **AUC ^(0-40)^ (nmol, geometric mean; 95% CI)** | **P-value (ANCOVA)** | **Lag time (h, mean ± SD)** | **Beta estimate,**  **P-value^a^** | **Absorption rate constant (h^-1^, mean ± SD)** | **Beta estimate, P-value^a^** |
| --- | --- | --- | --- | --- | --- | --- | --- | --- |
| rs893051 | Oxybenzone | homozygous minor | 1475.9; 914.56, 2689.6 | 0.117^b^ | 0.65 ± 0.2 |  | 0.28 ± 0.1 |  |
|  |  | heterozygous | 1068.4; 850.21, 1340.7 |  | 0.26 ± 0.2 | **–319, <2.2e-16** | 0.16 ± 0.04 | **–32.6, <2.2e-16** |
|  |  | homozygous major | 901.95; 689.04, 1163.3 |  | 0.19 ± 0.06 | **–354, <2.2e-16** | 0.18 ± 0.04 | **–21.4, <2.2e-16** |
|  | Pyrimethanil | homozygous minor | 1692.7; 1146.9, 2574.2 | 0.542^c^ | 0.69 ± 0.2 |  | 0.2 ± 0.06 |  |
|  |  | heterozygous | 1461; 1117.5, 1875.5 |  | 0.34 ± 0.08 | **–23.5, <2.2e-16** | 0.14 ± 0.05 | **–3.5, 0.001** |
|  |  | homozygous major | 1281.3; 873.83, 1817.1 |  | 0.29 ± 0.06 | **–27.6, <2.2e-16** | 0.16 ± 0.05 | –1.98, 0.05 |
|  | Pyrene | homozygous minor | 27.7; 20.3, 36.7 | 0.423^d^ | 1 ± 0.2 |  | 0.077 ± 0.009 |  |
|  |  | heterozygous | 28.6; 24.2, 34.5 |  | 1.2 ± 0.2 | **2.82, 0.007** | 0.074 ± 0.008 | **–2.49, 0.02** |
|  |  | homozygous major | 23.5; 17.7, 31.6 |  | 1.1 ± 0.2 | 1.44, 0.2 | 0.076 ± 0.007 | –0.14, 0.9 |
| rs2303067 | Oxybenzone | homozygous minor | 1207.2; 753.62, 1987.8 | 0.542^e^ | 0.63 ± 0.1 |  | 0.2 ± 0.04 |  |
|  |  | heterozygous | 1002.3; 825.84, 1239.7 |  | 0.22 ± 0.1 | **–347, <2.2e-16** | 0.2 ± 0.03 | 0.42, 0.7 |
|  |  | homozygous major | 1142.7; 874.28, 1513.4 |  | 0.62 ± 0.08 | **5.44, 2.1e-6** | 0.28 ± 0.03 | **14.8, <2.2e-16** |
|  | Pyrimethanil | homozygous minor | 1569.5; 1059.9, 2262.7 | 0.8^f^ | 0.21 ± 0.03 |  | 0.14 ± 0.04 |  |
|  |  | heterozygous | 1419.9; 1071.3, 1863.8 |  | 0.42 ± 0.06 | **88.4, <2.2e-16** | 0.15 ± 0.05 | 0.49, 0.6 |
|  |  | homozygous major | 1305.6; 889.89, 1900.8 |  | 0.77 ± 0.08 | **127, <2.2e-16** | 0.17 ± 0.03 | 1.88, 0.07 |
|  | Pyrene | homozygous minor | 27.3; 20.6, 36.2 | 0.933^g^ | 1.1 ± 0.3 |  | 0.077 ± 0.009 |  |
|  |  | heterozygous | 26.2; 21.6, 31.8 |  | 1.2 ± 0.2 | 1.76, 0.08 | 0.073 ± 0.007 | **–2.7, 0.01** |
|  |  | homozygous major | 28.3; 24.2, 33.4 |  | 1.1 ± 0.1 | **2.66, 0.01** | 0.084 ± 0.005 | **2.98, 0.005** |

1. Beta estimates and p-values are retrieved from the linear regression performed in Monolix, where they are deviations from “Homozygous minor”-genotype estimates. Bold indicates significance (p<0.05).
2. Adjusted R^2^ = 0.177. Statistically significant pairwise comparation between homozygous major and homozygous minor; p = 0.04
3. Adjusted R^2^ = 0.101
4. Adjusted R^2^ = 0.143
5. Adjusted R^2^ = 0.119
6. Adjusted R^2^ = 0.086
7. Adjusted R^2^ = 0.113

**Supplementary Table 3.** Area under the excretion curve (AUC_(0-40 h)_), lag time, and absorption rate by genotypes of rs893051 (*CLDN1*) and rs2303067 (*SPINK5*), adjusted for age, sex, body mass index (BMI), and FLG copy number variation (CNV) score.

| **Variant** | **Chemical** | **Genotype** | **AUC ^(0-40)^ (nmol, geometric mean; 95% CI)** | **P-value (ANCOVA)** | **Lag time (h, mean ± SD)** | **Beta estimate,**  **P-value^a^** | **Absorption rate constant (h^-1^, mean ± SD)** | **Beta estimate, P-value^a^** |
| --- | --- | --- | --- | --- | --- | --- | --- | --- |
| rs892051 | Oxybenzone | homozygous minor | 1475.9; 908.9, 2623.5 | 0.129^b^ | 0.59 ± 0.3 |  | 0.26 ± 0.04 |  |
|  |  | heterozygous | 1123.8; 851.4, 1457.8 |  | 0.1 ± 0.1 | **–23013, <2.2e-16** | 0.14 ± 0.03 | **–12.5, 1e-15** |
|  |  | homozygous major | 861; 668.7, 1111.5 |  | 0.023 ± 0.04 | **–33967, <2.2e-16** | 0.18 ± 0.03 | **–7.27, 6e-9** |
|  | Pyrimethanil | homozygous minor | 1692.7; 1151.4, 2558.7 | 0.551^c^ | 0.42 ± 0.2 |  | 0.22 ± 0.06 |  |
|  |  | heterozygous | 1422.4; 1085.1, 1830.8 |  | 0.48 ± 0.2 | **–215, <2.2e-16** | 0.19 ± 0.05 | **–1720, <2.2e-16** |
|  |  | homozygous major | 1224.1; 813.4, 1820.1 |  | 0.33 ± 0.06 | **–260, <2.2e-16** | 0.19 ± 0.04 | **–1130, <2.2e-16** |
|  | Pyrene | homozygous minor | 27.7; 20.1, 37.5 | 0.502^d^ | 1.07 ± 0.2 |  | 0.077 ± 0.006 |  |
|  |  | heterozygous | 27.6; 23, 32.8 |  | 1.16 ± 0.3 | –0.60, 0.55 | 0.072 ± 0.006 | **–20.9, <2.2e-16** |
|  |  | homozygous major | 23.7; 17.7, 31.8 |  | 1.09 ± 0.3 | –0.68, 0.50 | 0.074 ± 0.005 | **–14.8, <2.2e-16** |
| rs2303067 | Oxybenzone | homozygous minor | 1207.2; 518.9, 1535.5 | 0.131^e^ | 0.0014 ± 0.0007 |  | 0.14 ± 0.04 |  |
|  |  | heterozygous | 958.2; 767.4, 1162 |  | 0.079 ± 0.04 | **228915, <2.2e-16** | 0.17 ± 0.05 | **2.33, 0.02** |
|  |  | homozygous major | 1455.9; 927.3, 2579.5 |  | 0.031 ± 0.02 | **134931, <2.2e-16** | 0.2 ± 0.05 | **2.91, 0.006** |
|  | Pyrimethanil | homozygous minor | 1569.5; 703.7, 1827.1 | 0.592^f^ | 0.25 ± 0.04 |  | 0.26 ± 0.07 |  |
|  |  | heterozygous | 1359.2; 1026, 1780.9 |  | 0.42 ± 0.09 | **26743, <2.2e-16** | 0.29 ± 0.1 | 0.44, 0.7 |
|  |  | homozygous major | 1305.6; 888.8, 1876.6 |  | 0.65 ± 0.09 | **43256, <2.2e-16** | 0.33 ± 0.07 | **3.99, 0.0003** |
|  | Pyrene | homozygous minor | 27.3; 14.3, 44.4 | 0.891^g^ | 1.02 ± 0.3 |  | 0.08 ± 0.008 |  |
|  |  | heterozygous | 25.5; 21, 30.8 |  | 1.15 ± 0.4 | 2.2, 0.03 | 0.072 ± 0.006 | **–48.8, <2.2e-16** |
|  |  | homozygous major | 28.3; 24.2, 33.2 |  | 1.26 ± 0.3 | **3.9, 0.0003** | 0.085 ± 0.006 | **25.5, <2.2e-16** |

1. Beta estimates and p-values are retrieved from the linear regression performed in Monolix, where they are deviations from “Homozygous minor”-genotype estimates. Bold indicates significance (p<0.05).
2. Adjusted R^2^ = 0.232.
3. Adjusted R^2^ = 0.196
4. Adjusted R^2^ = 0.181
5. Adjusted R^2^ = 0.316
6. Adjusted R^2^ = 0.308
7. Adjusted R^2^ = 0.279


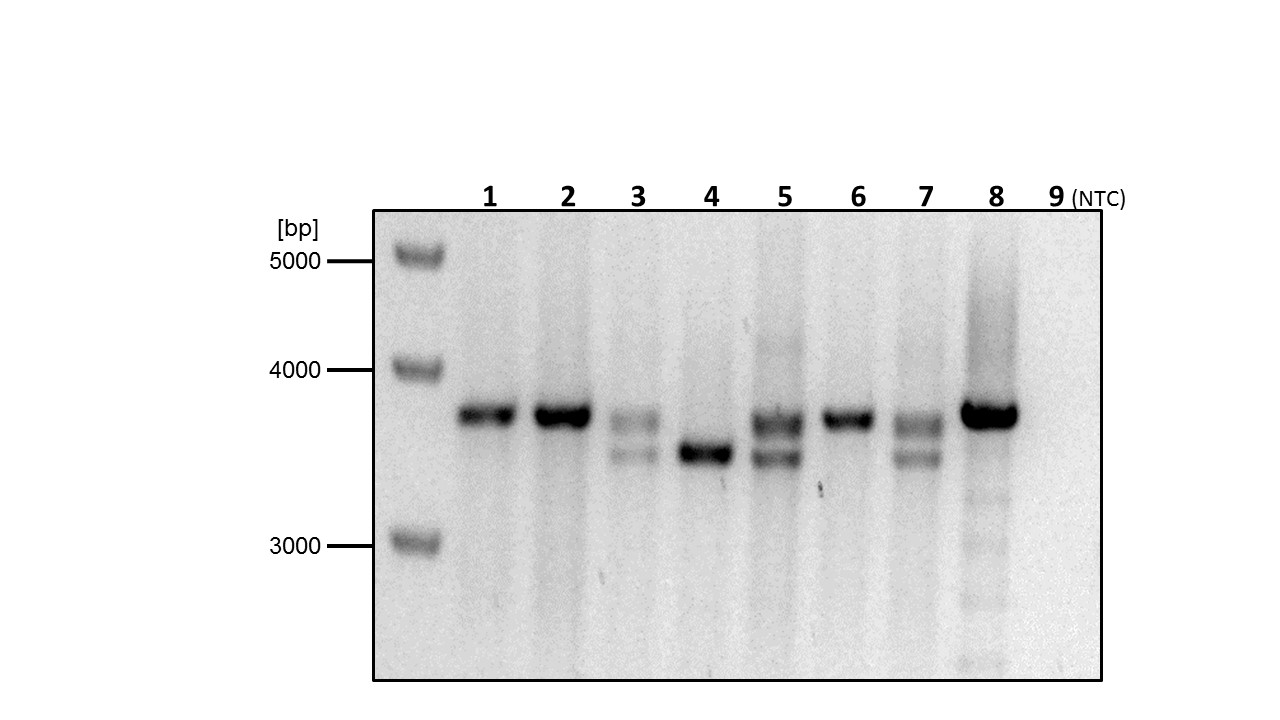


**Supplementary Figure 1.** *FLG2* CNV amplicons separated on a 1% agarose gel. The 5’ end sequence of *FLG2* exon 3 containing the variant deletion(s) was amplified from DNA samples using long-range PCR. The PCR products (1–9) were separated by size on a 1% agarose gel using gel electrophoresis at 100 V for 5 h 20 min and visualized with UV (302 nm) light. The upper gel bands represent larger amplicons of the *FLG2* sequence, approximately 3800 bp, while the lower gel bands are the shorter amplicons of 3600 bp. Their expected sizes were 3659 bp and 3204 bp, respectively. The absence of a lower gel band is interpreted as a homozygous genotype for *FLG2* (without the domain deletion(s)), as seen in samples 1, 2, 6, and 8. The absence of the upper gel band is interpreted as a homozygous genotype for *FLG2* (with the domain deletion(s)), as seen in sample 4. The presence of both the upper and lower bands is interpreted as a heterozygous genotype. A 1-kb Plus Ladder (Thermo Fisher Scientific) is shown on the left. NTC: negative control. Total sample size: N = 52.

# References

Altschul, S. F., Gish, W., Miller, W., Myers, E. W., & Lipman, D. J. (1990). Basic local alignment search tool. *J Mol Biol*, *215*(3), 403-410. <https://doi.org/10.1016/s0022-2836(05)80360-2>

Benson, G. (1999). *Tandem repeats finder: a program to analyze DNA sequences*. Nucleic Acids Research. <https://tandem.bu.edu/trf/run_web_submission>

Madeira, F., Pearce, M., Tivey, A. R. N., Basutkar, P., Lee, J., Edbali, O., Madhusoodanan, N., Kolesnikov, A., & Lopez, R. (2022). Search and sequence analysis tools services from EMBL-EBI in 2022. *Nucleic Acids Research*, *50*(W1), W276-W279. <https://doi.org/10.1093/nar/gkac240>

Rietz Liljedahl, E., Johanson, G., Korres de Paula, H., Faniband, M., Assarsson, E., Littorin, M., Engfeldt, M., Lidén, C., Julander, A., Wahlberg, K., Lindh, C., & Broberg, K. (2021). Filaggrin Polymorphisms and the Uptake of Chemicals through the Skin-A Human Experimental Study. *Environ Health Perspect*, *129*(1), 17002. <https://doi.org/10.1289/ehp7310>

Ye, J., Coulouris, G., Zaretskaya, I., Cutcutache, I., Rozen, S., & Madden, T. L. (2012). Primer-BLAST: a tool to design target-specific primers for polymerase chain reaction. *BMC Bioinformatics*, *13*, 134. <https://doi.org/10.1186/1471-2105-13-134>
